# Impact of LaZnFe_2_O_4_ supported NiWO_4_@D_400_-MMT@CMS/MMA nanocomposites as a catalytic system in remediation of dyes from wastewater

**DOI:** 10.1038/s41598-024-61565-7

**Published:** 2024-05-21

**Authors:** Safaa. R. Fouda, Salah A. Hassan

**Affiliations:** 1 Chemical Engineering Department, Higher Institute of Engineering and Technology, MNF-HEIT, Cairo, Egypt; 2 Chemistry Department, Faculty of Science, Ain Shams University, Abbassia, Cairo, 11566 Egypt

**Keywords:** Lanthanum zinc ferrite, Nickel tungstate, Montmorillonite, Nanocomposite, Adsorption, Cationic dye, Amphoteric dye, Environmental sciences, Chemistry, Materials science

## Abstract

Herein, a novel nanocomposite based on lanthanum zinc ferrite and nickel tungstate was created by incorporation between (MMT-jeffamine-400) nanoparticles (NPs), chloromethyl styrene as a binder and polymethyl methacrylate monomer using solution polymerization. The as-designed nanocomposites were employed to confiscate xylenol orange “X.O” as an acidic dye and rhodamine B “RhB” as “an amphoteric dye” from colored wastewater. The impact of several parameters such as solution pH, initial dye concentration, the effect of time, and the effect of temperature was explored. The consequences indicated that the pure organoclay had negligible adsorption while that composed of organoclay with PMMA@CMS-polymer incorporated with LaZnFe_2_O_4_@NiWO_4_ particles detached more than 90% for xylenol orange (XO) and 93% for “rhodamine B” molecules. Electrostatic interactions are the predominant factor in the adsorption of cationic and amphoteric adsorbates, as proven by zeta-potential measurement. Additionally, the adsorbent may be regenerate and utilized up to five times with good adsorption capabilities by adding sodium hydroxide. As a result, the removal can be effectively accomplished using the nanocomposite as an adsorbent. The actual and theoretical adsorption capacity values for both dyes at all doses were closely matched, which supported the adsorption kinetics data that fit the pseudo-first order rate model well. The adsorption data’s correlation values (0.995 for XO and 0.98 for RhB) indicated that both dyes’ Langmuir adsorption would perform well. Furthermore, the adsorption of XO and RhB dyes on the adsorbent is confirmed to be a viable reaction by the negative values of ΔGo. The enhanced adsorbent material for the removal of amphoteric and anionic dyes from waste water is the synthesized LaZnFe2O4 supported NiWO4@D400-MMT@CMS/MMA nanocomposites, which exhibits a reusability affinity of up to five cycles.

## Introduction

Nanotechnology plays a vital role in wastewater treatment due to the optimization of high porosity, hydrophobicity, and dispensability. Dyes, heavy metals, phenols, pesticides, hydrocarbons, and organic compounds are among the contaminants found in wastewater. Dyes are chief coloring agents in paper, leather, plastic, textile, pharmaceuticals, food and cosmetics industries. These companies release between 10 and 15% of their colors as effluents into the water system. As a consequence, it is a global challenge to provide safe water for drinking. To eliminate these pollutants from water, there are several techniques were used such as chemical precipitation, conventional coagulation, reverse osmosis, ion exchange, electrodialysis, electrolysis, and adsorption. Adsorption is the most powerful method for water purification. Not only depending on its cost but also the adsorption capacity of the adsorbent. Adsorption is the less costly method to eliminate both organic and inorganic waste from water^1–3^**.** For instance, a nanocomposite based on monodispersed silica nanoparticles incorporated nanocomposites of Gelatin and Psyllium was used for removal of brilliant green by 90%, and xylenol orange by 100%^4^**.** In order to overcome the drawbacks of the two processes mentioned above, researchers have combined them to create a brand-new method called adsorption-photocatalysis. Adsorption-photocatalysis is a cost-effective, energy-efficient method of detoxifying water that is also highly selective for certain pollutants. As a result, it is considered an environmentally benign therapy^5,6^**.**

Nanocomposites that contain nanoparticles improved their properties as have excellent capability, selectivity, and stability for water treatment. The use of advanced technology such as melting fluids, melting mixing, deposition layer by layer, in situ polymerization, electro-polymerization, and surface initiation polymerization has synthesized a wide range of conducting polymer nano-compounds.

The focus of recent research on polymer nanocomposite for the removal of water contaminants^7–11^**.** Clays and silicates are used as inorganic nanofillers due to their low cost, high aspect ratio, rich intercalation chemistry, high strength, stiffness, and thermal stability of polymers^12–15^**.** The size of clay layers dispersion and morphology found in the polymer matrix (intercalation, exfoliation, mixed intercalation). The possessions of the gas barrier are heavily influenced by exfoliation, accumulation, etc. High quality of exfoliation and desired platelet orientation remained a difficult task^16–18^**.** The organic adjustment of the interlayer gallery with organic ammonium, sulfonium, or phosphonium will increase the affinity between the clay and the polymer^19^**.** Many metals, metal oxides, and sulfides were presently used as water and wastewater disposal catalysts both in front and without light^20^**.** Catalytic degradation of various nitrogen-containing contaminants compounds dyes and organic residual compounds has been investigated^21^.

Due to their special physical and chemical qualities, spinel zinc ferrite (ZnFe_2_O_4_) nanoparticles have drawn particular interest from a wide range of industries. These include gas sensing, drug delivery, and magnetic resonance imaging (MRI), as well as possible uses in photocatalysis and other areas^22–30^**.** Particularly, there are many opportunities offered by nanoscale semiconductor particles with a range of shapes and appropriate surface areas. In contrast to alternative photocatalysts, iron oxides are abundant in nature and do no harm to the environment^31–34^**.** Han et al.^35^ argue synthesis of La_2_O_2_CO_3_/ZnFe_2_O_4_-reduced graphene oxide nanohybrid through a simple precipitation-hydrothermal method. When compared to pure rGO and ZF, LGZF shown a higher adsorption capacity. When it came to MB and RhB, for example, LGZF with a 1:1 mass ratio demonstrated the best adsorption efficiency at 97.3 and 94.2%, respectively.

This work presents the preparation and application of a unique magnetic LaZnFe_2_O4 supported NiWO4@D400-MMT@CMS/MMA nanocomposites nanohybrid for the first-ever improved removal of XO and RhB from aqueous solutions. The initial goal of this work was to comprehend the LaZnFe2O4@NiWO4@D400-MMT@CMS/MMA nanocomposites’ adsorption characteristics. For practical reasons, the suitability of several adsorbents for the elimination of organic dyes from surface waters was assessed. A number of operational factors were methodically examined, and the adsorbent’s surface and crystal structures were described. When the produced material was compared to D400-MMT@CMS/MMA nanocomposites alone, it showed an impressive adsorption ability on both x and RhB. Remarkably, the elimination of xo and RhB was found to benefit from the synergistic effects of LaZnFe2O4, NiWO4, and D400-MMT@CMS/MMA nanocomposites. Moreover, the presence of the distinctive La, Zn,Ni, and WO4 elements on D400-MMT@CMS/MMA nanocomposites suggests that π–π interaction, electrostatic attraction, hydrogen bonding, and surface complexation are viable adsorption processes.” While I worked with 50 ppm of dye concentration, all prior study concentrated on 25 ppm.

Our efforts in the current work were focused on improving the depolluting properties of clay nanocomposite by immobilizing LaZnFe_2_O_4_ nanoparticles in NiWO_4_@clay-Jeffamine-CMS-MMA nanocomposite systems. LaZnFe_2_O_4_ and NiWO_4_ nanoparticles have interacted with the CMS-MMA matrix as guest species. The study was prolonged to explore their removal efficiencies and mechanisms. The investigated interaction profiles toward hazardous pollutants in aqueous media, namely xylenol orange (XO) as an acidic dye and Rhodamine B “RhB” as “an amphoteric dye.

## Experimental details

### Chemicals and methods

Sodium montmorillonite (Na-MMT) with a cation exchange capacity (CEC) of ca.119 m.equiv./100 was supplied from Kunimine Industry Co. Japan, under trade name Kunipia-F. Jeffamine was obtained from Huntsman Corporation, Texas, USA: poly(oxypropylene) diamine **(D**_**400**_**)** having an average molecular mass of 400, Brookfield viscosity at 25ºC is 21 and the primary amine content is 4.3 meq/g. Chloromethylstyrene (CMS) [Vinyl benzyl chloride] was obtained from (Fluka) poly science, Inc., as a mixture of m-/p-isomers (30:60%) and used as supplied. Methylmethacrylate (MMA) was used as purchased from Aldrich. Cetyl-trimethyl ammonium bromide (CTAB), was obtained from BDH. Xylenol orange (XO) tetrasodium salt dye (90%) (XO; C_31_H_28_N_2_Na_4_O_13_S; MW = 760.58 g/mol), Rhodamine B dye (98%) (RhB; C_28_H_31_ClN_2_O_3_; MW = 479.02 g/mol) and other reagents used in this work were obtained from BDH Chemical Reagents Co. Ltd.

### Fabrication of LaZnFe_2_O_4_@NiWO_4_@D400-MMT@MMA-CMS nanocomposites

#### poly (Oxypropylene) amine intercalated montmorillonite (D400-MMT) organoclay.

MMT (15 g) was swelled in 1000 ml of hot distilled water continuously for 3 h at 60 °C, followed by stirring for several hours at room temperature. To the swollen Na-MMT, 6 g of D_400_ was added, while being constantly stirred, and the dispersion was still heated at 60 °C. The polymer was protonated by adding hydrochloric acid, and the pH was increased to 8 to encourage the ion exchange reaction. After then, the blend was stirred for a further 24 h. The precipitate was filtered and repeatedly rinsed with water until no chloride ions could be found in the filtrate by using silver nitrate (AgNO_3_). The product D_400_-MMT was dried at 60 °C to yield 13 g. The non-bonded material was detached by washing with (1:1) H_2_O/methanol.

##### Measurement of swelling ratios

The swelling data demonstrates that the intercalation of poly (Oxypropylene) “jeffamine-D400” amine in the interlayer of MMT has converted the mineral MMT’s hydrophilic character into a hydrophobic one. When organic solvents are intercalated, the interlayer area swells, increasing the basal spacing. The swelling of D400-MMT nanocomposite in several solvents, including toluene, benzene, DMF, EtOH, water, dioxane, and acetone. Similarly, the attraction between D-400 chain in the interlayer area and the organic solvent is thought to facilitate the penetration process of the organic solvent. The solvation effectiveness of the D400-MMT with solvent depends only on the nature of the onium cation intercalated in the MMT and the chemical structure of the solvent used. According to the swelling results, the hydrophilic nature of the mineral MMT has converted into a hydrophobic nature as a result of D-400 intercalation in the interlayer of MMT. Moreover, the substituted poly (Oxypropylene interacts more strongly with toluene, and this interaction controls how D400-MMT swells in non-polar solvents. To guarantee complete swelling, the enlarged hydrogels were removed after 72 h. The swelling ratio SR (g.g^−1^) was calculated using the following equation:

1 $${\text{SR = }}\frac{{\left( {{\text{W}}_{{1}}- {\text{ W}}_{{0}} } \right)}}{{{\text{W}}_{{0}} }}$$

where W_1_ = final weight after swelling and W_o_ is the initial weight.

The equilibrium swelling ratio SRe, significantly increased for DMF, going from 1890 to 140.7% for acetone. the highest ratio for dimethyl formamide, indicating that DMF is the sufficient solvent employed in the procedure.

#### Synthesis of polymethyl methacrylate (20%)-chloromethyl styrene as a binder (10%) @ jeffamine.400-montmorillonite (D400-MMT) (70%) nanocomposite

(D400-MMT) (7 g) was swelled in 5 ml DMF and 20 ml distilled H_2_O by stirring overnight for 24 h at room temperature under vigorous stirring. A solution of (2 g) methyl methacrylate and (1 g) chloromethyl styrene were dissolved separately in 5 ml DMF, and dropwise to (D400-MMT). The reaction mixture was stirred overnight at 60 ºC for 24 h. The product was filtered, washed several times with water and collected by a filter press, and dried at 80 °C in a vacuum oven for 10 h.

#### Synthesis of lanthanum doped zinc ferrite nanoparticles LaZnFe_2_O_4_

Zinc nitrate hexahydrate (ZnNO_3_)_2_.6H_2_O (0.297 g, 1 mmol), Iron (III) Nitrate Nonahydrate Fe (NO_3_)_3_.9H_2_O (0.789 g,1.95 mmol), and Lanthanum (III) nitrate hexahydrate La (NO_3_)_3_.6H_2_O (0.0216 g, 0.05 mmol) were dissolved separately in 30 ml propylene glycol. (0.364 g, 1 mmol) of cetyl trimethyl ammonium bromide was added to zinc nitrate hexahydrate solution. Another solution involves both ferric Nitrate and Lanthanum (III) nitrate hexahydrate added to the above solution. The final homogenous solution was kept stirring at 180 °C to form a gel, after that the product was calcinated at 800 °C^35,36^**.**

#### Synthesis of LaZnFe_2_O_4_@ NiWO_4_ @methylmethacrylate chloromethyl styrene (CMS)@montmorillonite Jeffamine (D400-MMT) “S_3_”

**Figure 1:**
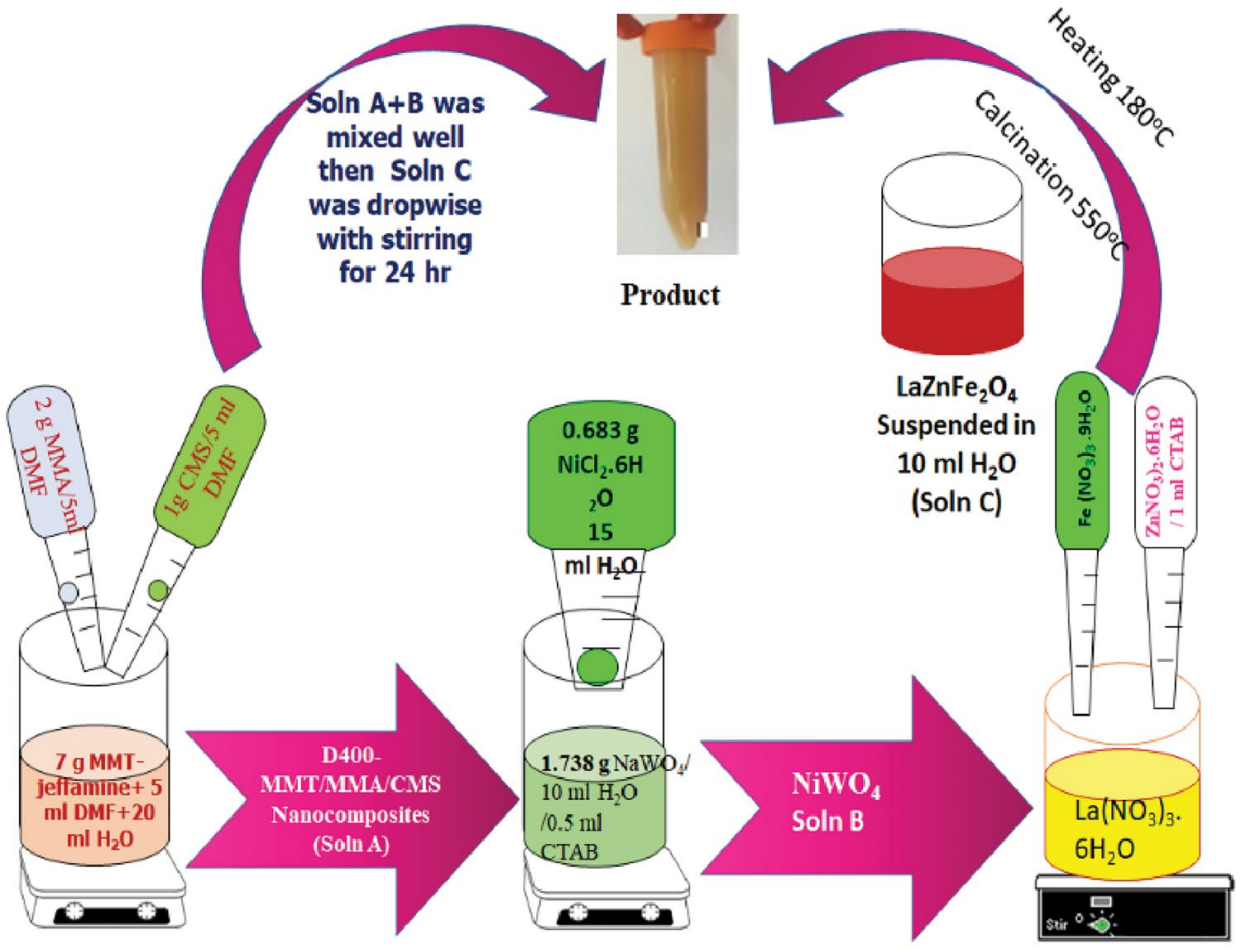
Schematic representation of the system used to LaZnFe_2_O_4_@NiWO_4_@D_400_-MMT@CMS/MMA nanocomposites.

LaZnFe_2_O_4_ (0.113 g) was suspended in 10 ml distilled H_2_O under vigorous stirring for 24 h **(soln B).** Soln B was added to the suspended polymer clay nanocomposite (soln A), leaving the mixture under magnetic stirring to form a homogeneous solution. 1.738 g of sodium wolframite NaWO_4_ dissolved in 20 ml distilled H_2_O. Cetyl trimethyl ammonium bromide solution (0.5 ml, 25%) was added with stirring for 1 h. 0.683 g of NiCl_2_.6H_2_O dissolved in 15 ml dist H_2_O was added to the earlier mixtures with stirring for 3 h, **(soln D).** Soln D was added to a mixture containing both **Soln A and Soln B**, the formed mixture was stirred at room temperature for 24 h, then **Soln C** was added dropwise. Leave homogenous suspension under sonication at room temperature for another 1 h (the total volume of solution is 110 ml). The product was washed with H_2_O several times, then ethanol 3 times, centrifuged, then dried under a vacuum at 80 °C for 24 h. Table 1 explains the exploration of the as-designed LaZnFe_2_O_4_@NiWO_4_@ CMS-MMA @ D400-MMT nanocomposites. Figure 1. demonstrates the schematic representation of the as-designed LaZnFe_2_O_4_@NiWO_4_@D_400_-MMT@CMS/MMA nanocomposites.

**Table 1:** Exploration of the as-designed LaZnFe2O4@NiWO4@ CMS-MMA @ D400-MMT nanocomposites.

Sample code	Nanocomposite name
S_o_	Montmorillonite clay modified by D400
S_1_	20% PMMA -10%CMS (as binder)-70%- D_400_-MMT
S_2_	NiWO_4_-20% PMMA -10%CMS-70%- D_400_-MMT
S_3_	LaZnFe_2_O_3_- NiWO_4_-20% PMMA -10%CMS-70%- D_400_-MMT

#### Adsorption studies

##### Effects of pH

Accordingly, a dye solution of 50 ppm/100 mL of water was subjected to an adsorption process by adding 0.05 g of S_1″_, S_2 ,_ and S_3″_. Adsorption studies were conducted at room temperature. The impact of pH was investigated by adjusting the initial pH of the dye solution from pH = 2 to pH = 10, using either 0.1 M NaOH or HCl. The removal efficiencies of the nanocomposites are examined using the following equation used to compute the percentage of dye removal:

2 $${\text{Removal \, efficiency }}\left( {\text{\% }} \right) = \frac{{C_{o} - C_{e} }}{{C_{o} }} \times 100$$

where, C_o_ and C_e_ (mg L^−1^) were the initial and the equilibrium concentrations of pollutant dye, respectively.

##### Effect of adsorbent dosage

To treat wastewater, the right dosage of adsorbent is essential. While other parameters, such as 50 mL of dye solution (50 mg L^−1^) with a pH value of 4 for XO and 8 for RhB, remained constant, the impact of adsorbent dosage was tested at a mass ranging from 10 to 80 mg at intervals of 10 mg.

##### Equilibrium isotherms and kinetics

The adsorption isotherms were studied with initial dye concentrations of 7.5, 15, 25, 50,100, 150, 200, and 300 mg/L. By interacting 0.05 g of sorbent with an aqueous solution, centrifugation, and then analyzing. The kinetic study was carried out by injecting 50 mg/L dye solutions, the optimum pH for removal XO is 4, while for RhB pH is equal to 8 were agitated with 0.05 g of adsorbent at room temperature for predetermined intervals of time. The thermostatic shaker with 200 rpm for 24 h was used to achieve equilibrium at various temperatures of 292, 298, 308, and 318 K. Samples were filtered through, and the residual dye concentration was determined using a UV–Vis spectrometer. The following equation was used to determine sorption capacity (q) which will be qt in removal kinetics studies and qe in case of equilibrium sorption experiments, were calculated as-

3 $${\mathbf{Sorption \, Capacity }}\left( {\mathbf{q}} \right){\mathbf{ = }}\left( {{\mathbf{C}}_{{\mathbf{O}}} {\mathbf{ - C}}_{{\mathbf{e}}} } \right)\frac{{\mathbf{V}}}{{\mathbf{m}}}$$

where C_o_ is the initial concentration of target compounds before adsorption.

(mg L^−1^), C_e_: is the equilibrium concentration of designated analytes after adsorption (mg L^−1^), V is the aqueous solution volume (L), m is the adsorbent dosage (g), and q_e_ is the adsorption capacity at equilibrium (mg/g).

The q_e_/C_e_ ratio yields the distribution coefficient **K**_**d**_.

4 $${\mathbf{K}}_{{\mathbf{d}}} {\mathbf{ = }}\frac{{c_{0} - c_{e} }}{{c_{0} }} \times \frac{V}{m}$$

### Characterization of the adsorbent

An X-ray diffractometer (XRD) was used to investigate the structure of the adsorbent using Cu Kα (λ = 0.1542 nm) radiation (D8 Advance X-ray diffractometer). The XRD was operated over a 2Θ range of 5–90, It was possible to learn more about the intercalation or exfoliation of the OMMT clay. The morphological characteristics were examined using a transmission electron microscope (TEM) and were made using a JEM-1011 (Japan) with an accelerating voltage of 100 kV. The samples were prepared by insertion of a copper TEM grid in the sample suspension and dried at room temperature. FT-IR Spectra were carried out using the KBr disc and recorded on the JASCO spectrophotometer model FT/IR-4100 Technique in the 4000–400 cm^-1^ range of wave numbers. The surface morphology of clay nanocomposites was studied using field-emission scanning electron microscopy (FE-SEM. On an LEO, Zeiss SEM, and FE-SEM pictures with energy dispersive X-ray analysis (EDX) were acquired. To examine the thermal characteristics of samples, thermogravimetric analyses (TGA) were performed using a Rheometric Scientific TGA1500 (Piscataway, NJ). Studies were carried out using 1–1.5 mg samples heated at 10 C/min from 15 to 900 °C under an inert atmosphere of nitrogen. The average zeta potential (ζ. av, mV) is determined by pHpzc for the 25 °C suspensions of the samples under investigation in water dispersed.

### Ethical approval

This article does not contain any studies involving animals performed by any authors. Also, this article does not contain any studies involving human participants performed by any authors.

## Results and discussion

### Characterization of the adsorbents

#### XRD analysis

**Figure 2:**
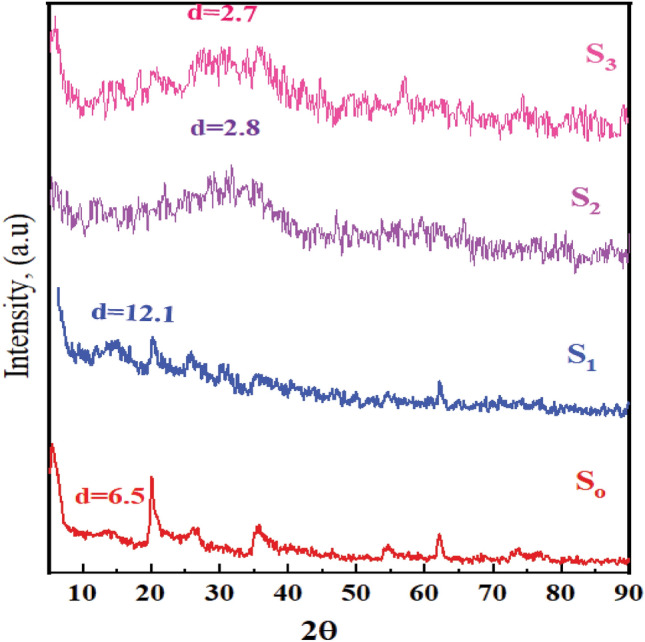
X-ray diffraction pattern of OMMT, MMA-CMS@ D_400_-MMT, NiWO_4_@ CMS-MMA@Jeffamine 400-MMT nanocomposite **(S**_**3**_**)** and LaZnFe_2_O_4_@Ni@CMS-MMA@ D_400_-MMT nanocomposites **(S**_**4**_**).**

The degree of clay intercalation and/or exfoliation in the polymer matrix has frequently been determined using X-ray diffraction (XRD). The wide-angle X-ray diffraction patterns of the OMMT and the PMMA-CMS@OMMT are displayed in Fig. 2 (**S**_**2**_). For the OMMT clay sample, the periodicity in the direction of (001) corresponds to the crystalline peak at 7.05 (d-spacing = 6.5 Å**)**^36^**.**

Most of the clay sheets in the polymer matrix have been exfoliated, as shown by the lack of a prominent peak in the XRD pattern for S_2_^37^**.** The individual diffraction peak of the clay interlayer vanished, showing the creation of the exfoliated structure. In addition to an expansion of the clay galleries by inserting polymer to become 12.14 Å. Subtracting the thickness of the silicate layer with D_400_ (6.5 Å) from the observed d_001_ spacing produces the amount of polymer within the interlamellar space 5.64 Å. This increase in the interlayer distance is the evidence that polymer has been successfully interring into the interlayer of MMT modified by **D-400.**

After hybridization with LaZnFe_2_O_4_, The XRD has a limit for analysis of other peaks that may lie in the broad peak at 2Θ = (20–40). These results also prove that metal entered the inter-gallery space of organoclay confirming the exfoliation structure with amorphous structure.

#### FT-IR analysis

**Figure 3:**
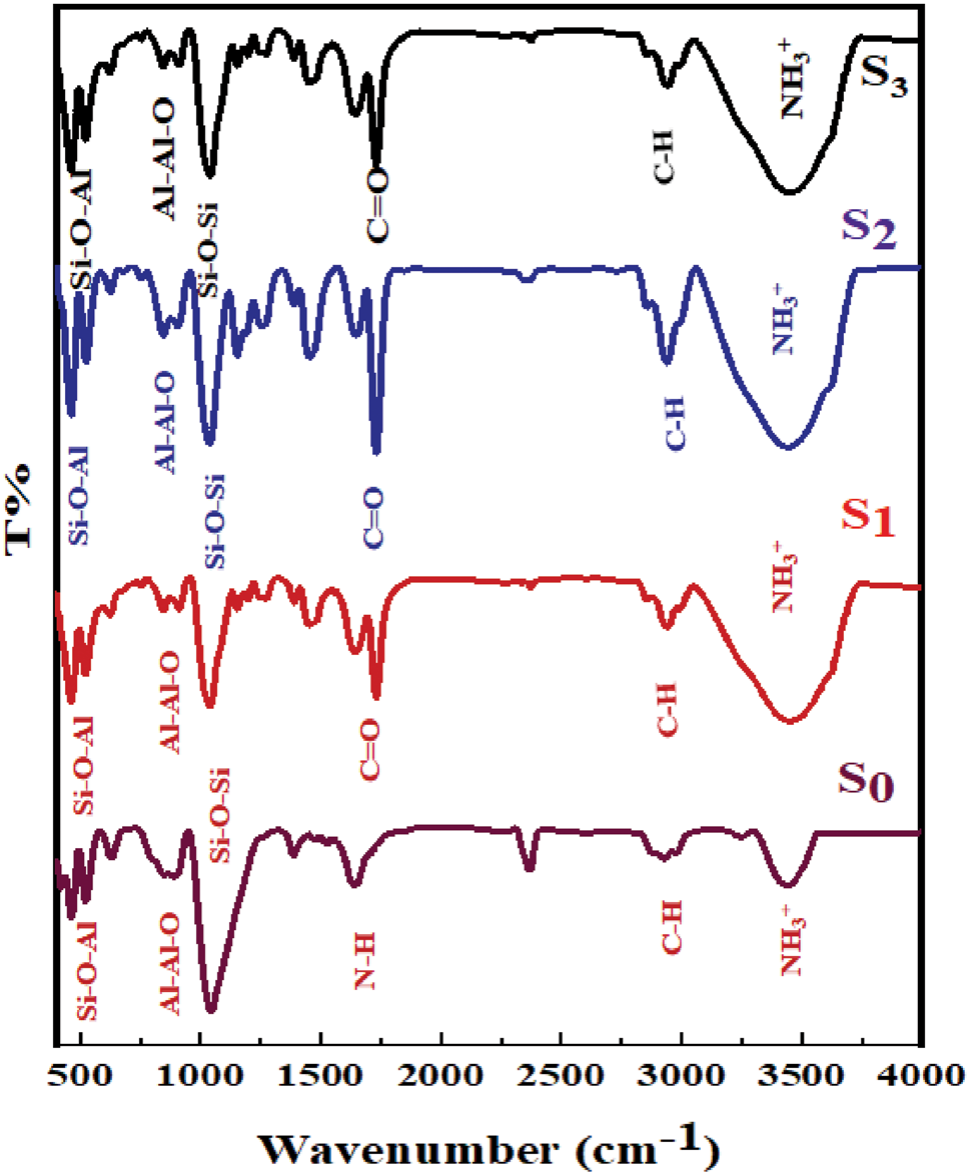
FT-IR spectra of OMMT, MMA-CMS@ D_400_-MMT, NiWO_4_@ CMS-MMA@Jeffamine 400-MMT nanocomposite **(S**_**3**_**)** and LaZnFe_2_O_4_@Ni@CMS-MMA@ D_400_-MMT nanocomposites **(S**_**4**_**).**

A chemical substance’s IR spectrum serves as a fingerprint for identification. The FTIR spectra from **(S**_**o**_**)**, **(S**_**1**_**)** as well **(S**_**2**_**)** and **(S**_**3**_**)** are depicted Fig. 3. The FTIR spectrum of (**S**_**o**_**)** demonstrated a stretching band at 1033 cm^−1^, assigned to the symmetrical Si–O-Si band [υ (Si–O-Si)]. The weak bands at 864 cm^-1^ and 530 cm^-1^ are considered to arise from [δ (Al-Al-O)] and [δ (Si–O-Al)], respectively. The typical bands correspond to the asymmetrical and symmetrical vibration of N–H in NH_3_^+^at [υ = 3435–3207 cm^−1^. The absorption bands at ~ 1617, 1395 cm^−1^ belong to the asymmetrical and symmetrical bending of N–H in NH_3_^+^ confirming the electrostatic attraction between Jeffamine and layered silicate**)**^38^**.**

However, for **(S**_**1**_**)** nanocomposite, the peaks at 1395 and 1450 cm^−1^ assignment to CH_3_ asymmetrical bending of epoxy. The sharp peaks at 1741 cm^−1^ are assigned to the (C = O) vibration of the carbonyl group. The weak peak at 828 cm^−1^ is related to the aromatic ring bending of chloromethyl styrene. The peak at 2961 cm^−1^ assigned to aliphatic C-H stretching which becomes more pronounced in **(S**_**2**_**)** nanocomposite and **(S**_**3**_**)** nanocomposite. Two differences are readily apparent from a comparison of IR spectra. (i) Peaks between 3428 cm^−1^ become stronger and wider, and (ii) the appearance of the peak at 2913 cm^−1^ belongs to aliphatic C-H stretching become more pronounced.

#### TEM results

**Figure 4:**
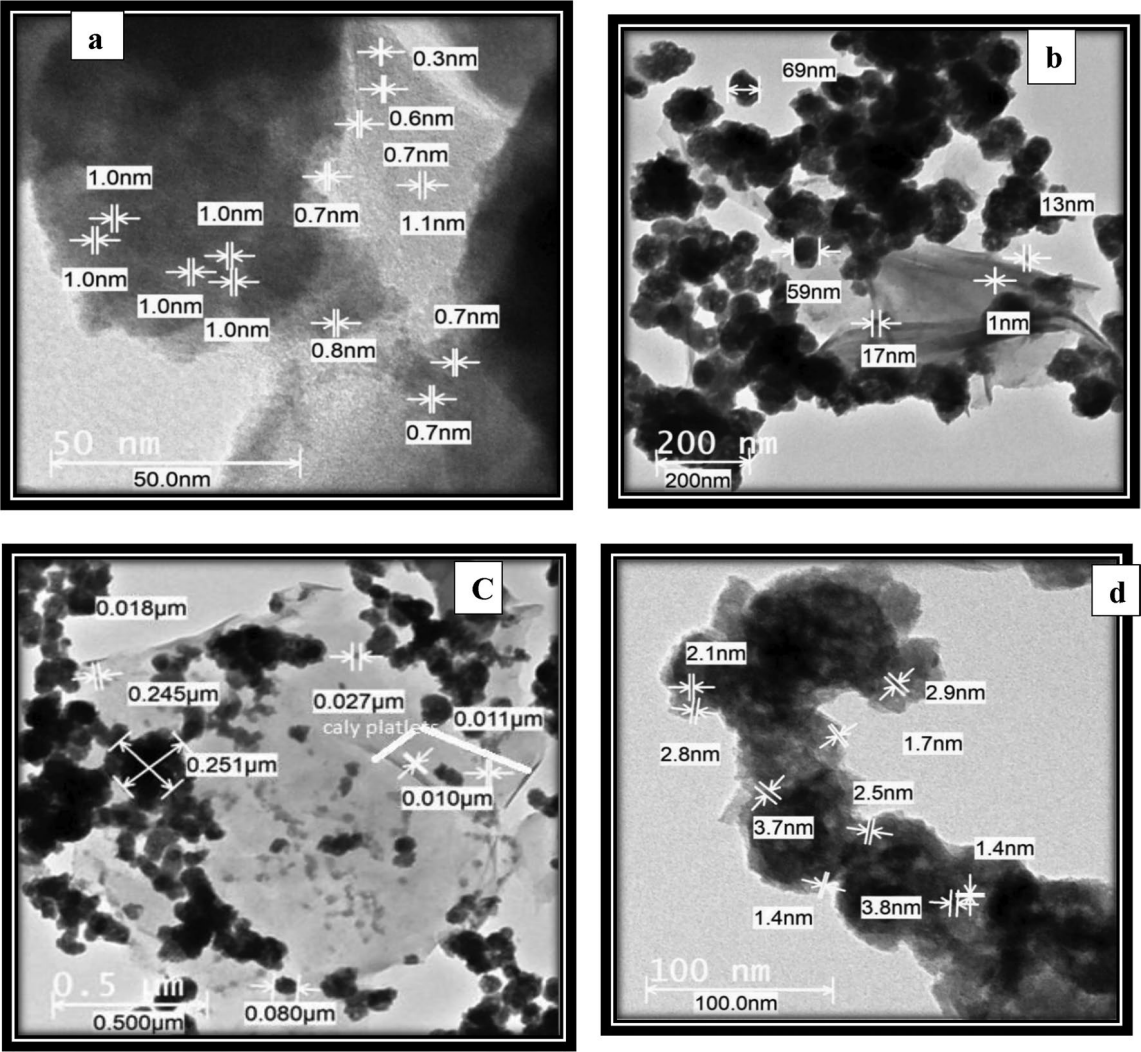
TEM micrographs: (**a**) Montmorillonite modified by poly(Oxy propylene) (OMMT), (**b**) 20% PMMA -10%CMS-70%- D_400_-MMT “S_1”_, NiWO_4_-20% PMMA -10%CMS-70%- D_400_-MMT “S_2_” and LaZnFe_2_O_3_- NiWO_4_-20% PMMA -10%CMS-70%- D_400_-MMT “S_3_^”^ nanocomposites. (**c**) NiWO^4^-20% PMMA-10%CMS-70%-D_400_-MMT, (**d**) LaZnFe_2_O_3_-NiWO_4_-20% PMMA-10%CMS-70%-D_400_-MMT.

The TEM image of OMMT was taken to better understand how poly (Oxy propylene) as surfactant dispersed inside the clay matrix. This picture is displayed in Fig. 4a. The size of some stacks appears to be around 0.7–1.1 nm, due to the presence of two amino groups in poly(Oxy propylene). These functional groups can establish hydrogen bonds with the silicate hydroxylated edge groups, resulting in a strong connection between the matrix and silicate layers. Figure. 4b describe the TEM of 20% PMMA -10%CMS-70%- D_400_-MMT “S_1_) which clarifies OMMT sheets as black strips. These strips are nicely distributed in the nanocomposite, demonstrating also that the majority of silicate layers are in the exfoliated structure. The clay platelets are filled with long-term polymers agglomerates. This is consistent with the conclusion drawn from XRD^23^**.** Figure. 4cThe coupling of nickel tungstate with the as-designed MMA-CMS@D_400_-MMT nanocomposite causes the clay platelets to become thinner and longer, much more separated. On the other hand, the TEM image of the as-synthesized LaZnFe_2_O_4_@NiWO_4_@MMA-CMS@D400-MMT nanocomposite in Fig. 4d shows that the incorporation of LaZnFe_2_O_4_NPs particles with NiWO_4_@MMA-CMS@D400-MMT are tending to exist in a uniform distribution mode, highly extended all over through the polymer clay colonies, as represented by the small dotes ranged from 1.4 to 3.8 nm.

#### SEM and EDX results

**Figure 5:**
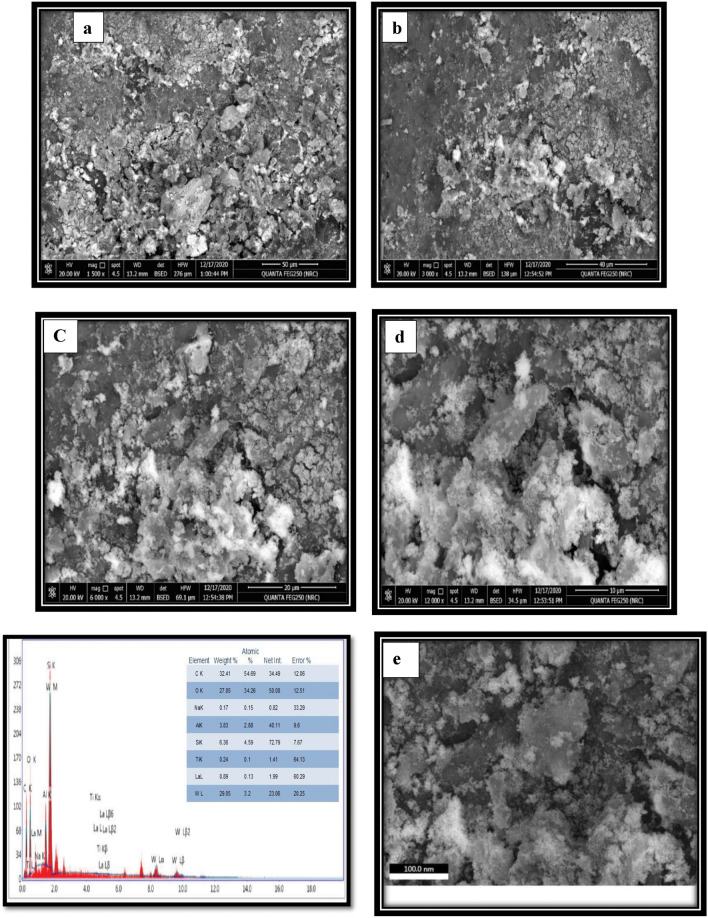
SEM micrographs (**a**) 20% PMMA -10%CMS (as binder)-70%-D_400_-MMT “S1”, (**b**) NiWO_4_-20% PMMA -10%CMS-70%-D_400_-MMT “S2” (**c**,**d**) LaZnFe_2_O_3_-NiWO_4_-20% PMMA -10%CMS-70%-D_400_-MMT nanocomposites “S3”, and (**e**) EDX “S3” nanocomposite.

On the other hand, the SEM image of LaZnFe_2_O_4_@ NiWO_4_ with organoclay 70% in the CMS-MMA matrix (Fig. 5a–d) appears in the flaky structure with irregularly shaped particles, also exhibits significant surface abrasion and compactness. The **(S**_**3**_**)** surface showed only a small number of cavities as depicted in Fig. 5c,d ^39^**.** These characteristics are crucial for sorption processes. The energy dispersive X-rays (EDX), Fig. 5e was employed to determine chemical composition using the EDX spectrum of the sample, It is clear that the main minerals in sodium montmorillonite clay are alumina (Al_2_O_3_), silica (SiO_2_), sodium, and titanium with minor amounts and no impurities. According to the montmorillonite’s EDX profile, the amounts of Si and Al are 9.97% and 17.18%, respectively. The proportions of other metals, such as K, Na, Ca, Mg, O, and Fe, are 0.41%, 0.32%, 0.49%, 0.79%, and 42.12%, respectively^40^**.**

Table 2. shows that after acid activation, a change in the chemical composition of sodium montmorillonite clay has taken place. One could say that acid treatment changes the structure of montmorillonite. Figure. 5e shows that the sharp peaks in the EDX spectrum were found to be caused by the elements C (32%), and O (27%). These substances will result in charges on the clay-exfoliated polymer’s surface and electrostatic forces that will attract the dyes in solution to the sample^41^**.** The fillers that were added to the polymer during processing to improve its photocatalytic characteristics may be the cause of the presence of La and W.

**Table 2:** Thermal analysis data of 20% PMMA -10%CMS-70%- D_400_-MMT “S_1”_, **(b)**NiWO_4_-20% PMMA -10%CMS-70%- D_400_-MMT, and LaZnFe_2_O_4_@ NiWO_4_@ CMS-MMA @70% wt D_400_-MMT nanocomposite.

*Sample*	TGA data						
	DTR						
	T_0.01_	T_0.05_	T_0.1_	T_i_	T_f_	Wt loss(%)	*Char yield(%)*
***S***_***1***_	25	95.7	251.7	99	893	84.6	15.4
***S***_***2***_	27.5	185.7	248	175	895	52.4	47.6
***S***_***3***_	27.6	92	251	188	895	47.2	52.8

#### Zeta potential measurements of LaZnFe_2_O_4_@ NiWO_4_@ CMS-MMA @70% wt D_400_-MMT nanocomposite

**Figure 6:**
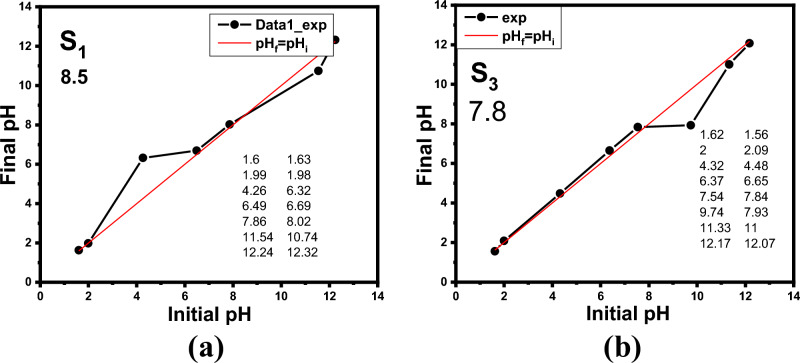
Zeta potential of (**a**) 20% PMMA -10%CMS-70%- D_400_-MMT “S_1”_, (**b**) LaZnFe_2_O_3@_NiWO_4_-20% PMMA -10%CMS-70%- D_400_-MMT nanocomposites as a function of pH.

The zeta potential is frequently viewed as a distant consequence of the particles’ surface charges. The absolute zeta potential value of S_1_ nanocomposite was lower than that of S_3_ nanocomposite, indicating that the modification reduces and increases surface charge. The measured values of ƹ-potentials as a function of pH in the 1–12 range for S_1_ nanocomposite was lower and S_3_ nanocomposite are positive, with an isoelectric point at 8.5 and 7.7 as demonstrated clearly (Fig. 6). This minor decrease of zeta potential is due to the increase of electrostatic strength of the suspension involved lanthanum zinc ferrite and intermolecular interactions^42^**.**

#### Thermogravimetric analysis of LaZnFe_2_O_4_@ NiWO_4_@ CMS-MMA @70% wt D_400_-MMT nanocomposite

**Figure 7:**
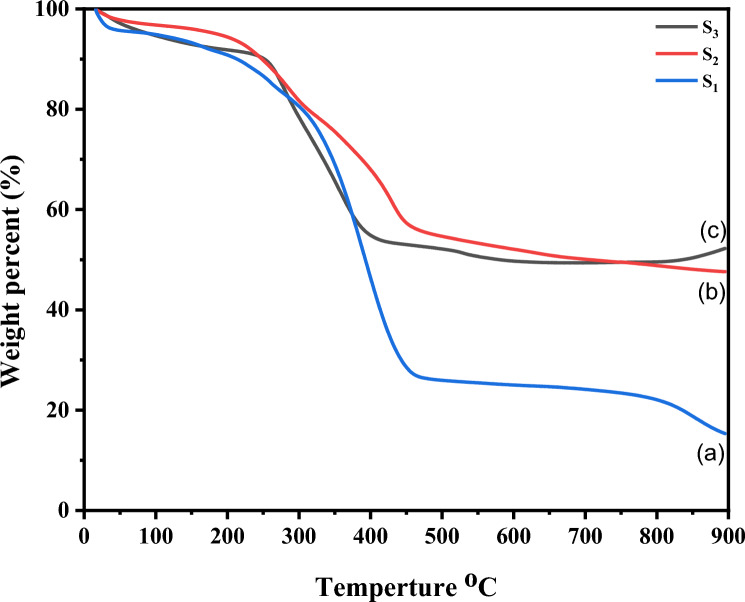
TGA Thermogram of (**a**) 20% PMMA -10%CMS-70%- D_400_-MMT “S_1”_, (**b**) NiWO_4_-20% PMMA -10%CMS-70%- D_400_-MMT, and (**c**) LaZnFe_2_O_4_@ NiWO_4_@ CMS-MMA @70% wt D_400_-MMT nanocomposite.

A useful tool for analyzing the thermal stability and thermal degradation behavior of materials is thermogravimetric analysis (TGA). MMT-D400 and CMS-MMA polymer nanocomposite demonstrated three mass losses, which occurred at 18–54 °C and 80 °C–468 °C, as shown in Fig. 7. The first mass loss is due to the release of water absorbed at the sorbents’ surface and results in a weight loss of around 4–5 wt.%. The second degradation step has a mass loss to be 69 wt.%. The third phase (up to 450 °C) reveals the total weight loss is 85 wt.%^43^**.**

The presence of nickel tungstate has a significant impact on sorbent stability. When compared to S_1_ as reference material, the inclusion of nickel tungstate effect on the degradation profile shows three major stages of weight loss, Water release (approximately 6% weight loss, at a maximum temperature of roughly 196 °C) is the first stage, which occurs below 196 °C. Weight loss is close to 42 wt. % in the range of 210–455 °C. The third phase (up to 461 °C) reveals a moderate weight loss (the total weight loss is about 52%).

The presence of **LaZnFe**_**2**_**O**_**4**_ has a significant impact on the sorbent stability. When compared to the reference material, the inclusion of **LaZnFe**_**2**_**O**_**4**_ affected the degradation profile to occur in two stages the weight loss is reduced to about 48%). Table 2 displays the TGA data including the temperatures at 1%, 5%, and 10% means thermal decomposition at (T_**0.01**_), (T_**0.05**_), and (T_**0.1**_), respectively.

## Adsorption process

Three distinct types of nanocomposites by improvement nanocomposites were prepared for the research of adsorption capabilities. These nanocomposites were investigated under a variety of adsorption circumstances, such as different contact periods and dye-starting concentrations.

### Optimization of solution pH for photo-adsorption study

**Figure 8:**
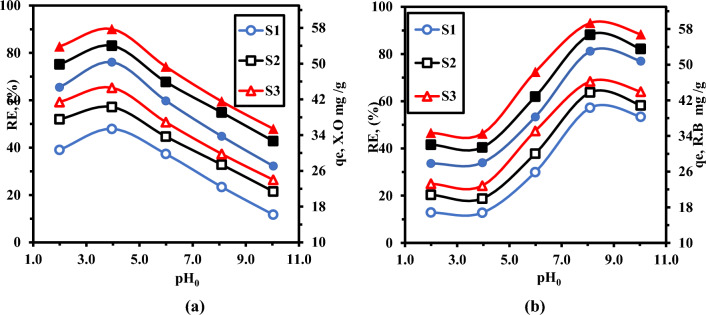
The effect of (a) the initial solution pHs on the removal efficiencies of (**S**_**1**_**)** 20% PMMA -10%CMS-70%- D_400_-MMT “S_1”_, (**S**_**2**_**)** NiWO_4_@ 20% PMMA -10%CMS-70%- D_400_-MMT, and ((**S**_**3**_**)** LaZnFe_2_O_4_@ NiWO_4_@ CMS-MMA @70% wt D_400_-MMT catalyzed systems (**a**) against the removal of XO (**b**) against the removal of R.B dye.

The first investigation was directed to choose the best-optimized condition. The investigation into the sorption effectiveness of xylenol orange (XO) as an acidic dye and rhodamine B (RhB) as an amphoteric dye onto the designed nanocomposite **“S**_**1**_**”**, **“S**_**2**_**”**, and **“S**_**3**_**”** nanocomposites as a function of pH (2–10) is shown in Fig. 8. pH has a considerable impact on the surface charge density of the adsorbent and dye speciation. The optimum pH for detachment of xylenol orange (XO) is 4, while for rhodamine **B** pH is equal to 8. It is clear from the data that the as-synthesized **(S**_**3**_**)** achieved the highest xylenol orange (XO) and Rhodamine B (RhB) removal efficiency (90.10%) and 93.15 when compared to S_1_, and S_2_ nanocomposites respectively^44^**.** However, employing a 50 mg/L XO and RhB solution, only 76.1% and 81.23% removal efficiency were achieved for the S_1_ nanocomposite, respectively. S_1_ was used as a reference sample. the effectiveness of **S**_**1**_ nanocomposite of XO removal gradually fails as pH rises, while the opposite trend is achieved for RhB.

It is necessary to investigate a variety of mechanisms, including electrostatic attraction/repulsion, and chemical interaction which seem to be in control of adsorption on adsorbent surfaces. Due to the increased concentration of hydronium ions (H^+^) at low pH, the clay surface becomes positively charged, boosting the propensity for the negatively charged COO^-^ at XO to bond on the clay without competing for the adsorption sites. As the pH is amplified, more OH becomes dissolved in the solution, competing with the xylenol orange(XO) for adsorbent active sites and causing low xylenol orange(XO) elimination at raised pH. These findings led to the decision to conduct all further studies carried out at pH 4. By increasing pH from 2 to 4, XO adsorption capacity for S_1_, S_2_, and S_3_ increased from 30.70, 37.54, and 41.40 mg/g to 35.40, 40.30, and 44.62 mg/g, respectively. Adsorption capacity gradually decreases as pH increase from 4 to 10.

Rhodamine B obeys the opposite trend of xylenol orange (XO). All the following tests were carried out at pH 8. It was found that the degree of ionization and speciation of the adsorbate, as well as the surface charge of the adsorbent, are all strongly influenced by the pH of the solution. RhB ‘s adsorption capacity on S_1_, S_2_, and S_3_ nanocomposites increased from 16.82, 20.80, and 23.28 mg/g to 40.34, 43.79, and 46.26 mg/g, respectively. Slightly decrease in adsorption capacity was achieved from 40.34, 43.79, and 46.26 mg/g to 38.32, 40.88, and 43.96 mg/g with an additional pH increase from 8.0 to 10.0.

More OH^-^ will be accessible at higher lower pH levels, enhancing the electrostatic interaction between negatively charged adsorption sites and positively charged dye cations and increasing dye adsorption. The strong electrostatic interaction between the negative surfaces of nanocomposite and R.B dye cations causes the high adsorption capacity. The attraction between cationic dye molecules and the excess hydroxyl ions causes a minor drop in adsorption at pH levels of 10.0. Owing to the chemical interaction that occurs between RhB dye and LaZnFe_2_O_4_@NiWO_4_@D400-MMT@MMA-CMS nanocomposites, the cationic dye still significantly adsorbed at pH = 8^45^**.** Importantly, electrostatic interactions excluding van der Waals are primarily responsible for the increased adsorption of amphoteric dye at high pH (and cationic dyes at low pH).

### Optimization of nanocomposites dosage

**Figure 9:**
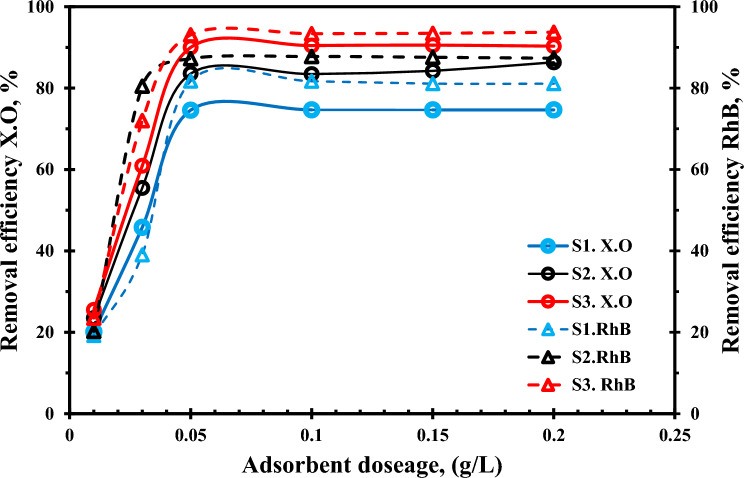
Effect of adsorbent dosage of (**S**_**1**_**)**, (**S**_**2**_**),** and ((**S**_**3**_**)** nanocomposites adsorption of XO. (pH_o_: 4.0, C_o_: 50 ppm, SD: 0.5 g/l, temperature: 25 °C), and RhB (pH_o_: 8.0, C_o_: 50 ppm, SD: 0.5 g/l, temperature: 25 °C).

For a given initial concentration of adsorbate in a solution, the solid adsorbent’s capacity is typically determined by the influence of the adsorbent mass. A significant factor in the color removal process was the dosage of the adsorbent. Figure 9 displays a plot of dye removal (%) vs adsorbent dosage (g L^-1^). As the dosage was increased, the amount of adsorption also increased. For XO and RB, the dosage above 0.05 g L^-1^ 0.2 did not significantly alter the adsorption effectiveness; instead, the dosage of 0.05 g L^-1^ was determined to be the optimum amount for adsorption at each pH.

### Adsorption kinetics

**Figure 10:**
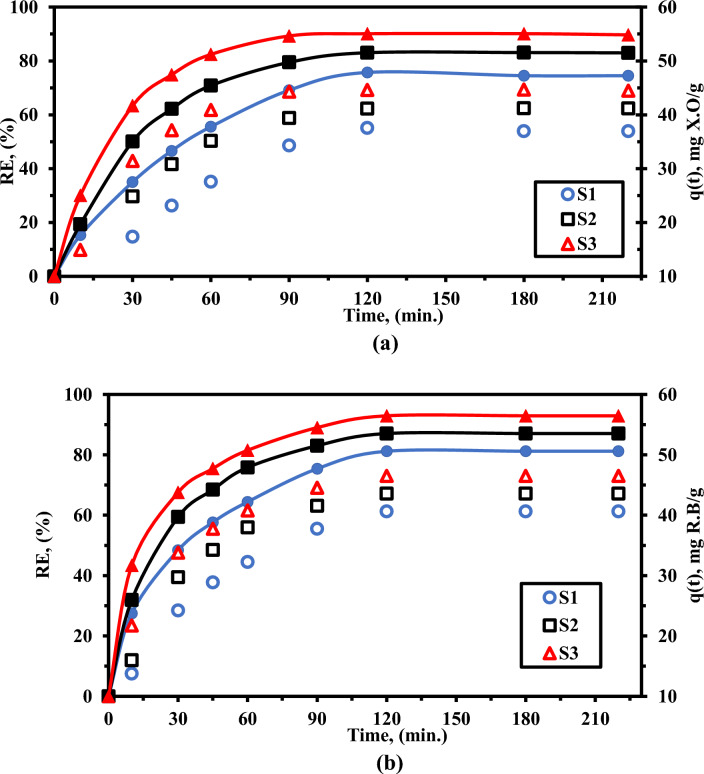
Shows the impact of adsorption time on the ability of (**S**_**1**_**)**, (**S**_**2**_**),** and ((**S**_**3**_**)** nanocomposites for XO. and RhB (**a**) Experiments on the adsorption of XO. (pH_o_: 4.0, C_o_: 50 ppm, SD: 0.5 g/l, temperature: 25 °C), (**b**) Experiments for RhB adsorption: (pH_o_: 8.0, C_o_: 50 ppm, SD: 0.5 g/l, temperature: 25 °C).

In a batch experiment, 100 mL Erlenmeyer flasks were used to carry out the adsorption of XO and RhB in aqueous solution on the as-prepared LaZnFe_2_O_4_ supported NiWO_4_@D400-MMT@CMS/MMA nanocomposites. The shaker was thermostated and the shaking speed was set to 150 rpm. At a temperature of 25 degrees Celsius, Fig. 10a,b demonstrated the impact of contact time on the quantity of removal of rhodamine B (RhB) as an amphoteric dye and xylenol orange (XO) as an acidic dye. At varying starting concentrations of xo and RhB, the LaZnFe_2_O_4_ supported NiWO4@D400-MMT@CMS/MMA nanocomposites exhibit the effects of contact duration on the quantity of XO and RhB adsorbed (Fig. 10a.b). Because there were many available active sites on the surface of the MMT@CMS/MMA nanocomposites during the initial adsorption stage, the adsorption rates during the first 30 min were quite rapid under all of the studied XO concentrations; subsequently, they became slower and consequently reached a plateau with the increase in contact time. More significantly, at the initial xo concentrations of 50 mg L^−1^, the removal efficiencies for S3, S3, and S1 were found to be 90%, 83%, and 74% during the adsorption process within 220 min. At the starting RhB concentrations of 50 mg L^−1^, the elimination efficiencies for RhB were determined to be 93%, 87%, and 81% for S_3_, S_2_, and S_1_.

**Figure 11:**
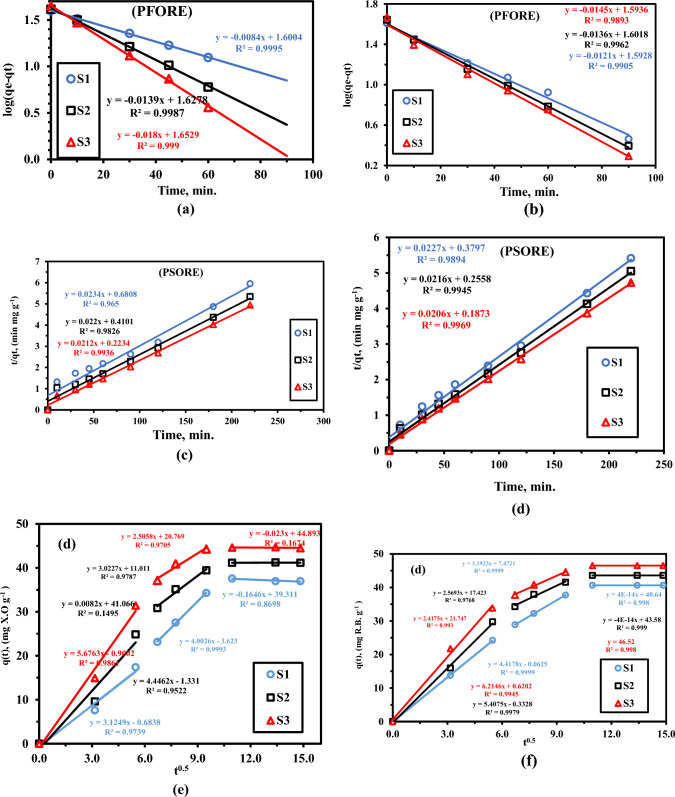
(**a-b**) models of pseudo-first order, (**c,d**) pseudo-second order kinetics, and (e,f) interparticle diffusion for the adsorption of dyes by (**S**_**1**_**)**, (**S**_**2**_**)** and ((**S**_**3**_**)** nanocomposites (In these studies, 0.05 g of nanocomposite beads were submerged in 100 mL of dye solutions (50 ppm, at room temperature).

This result illustrates the initial concentration-dependent adsorption performance of XO and RhB, as well as the outstanding efficiency of LaZnFe_2_O_4_@NiWO_4_@D400-MMT@CMS/MMA nanocomposites for XO and RhB removal in aqueous solutions. Pseudo-first- and pseudo-second-order kinetic equations were used to simulate the experimental data of XO and RhB adsorption on the LaZnFe_2_O_4_@NiWO_4_@D400-MMT@CMS/MMA nanocomposites in order to study the properties of the adsorption process as depicted in Fig. 11^46^.

5 $${\text{log}}\left( {qe - qt} \right) = \log q_{e} - \frac{{k_{1} t}}{2.303}$$

According to the pseudo-second-order model^31^**,**

6 $$\frac{t}{{q_{t} }} = \frac{1}{{k_{2} q_{e}^{2} }} + \frac{t}{{q_{e} }}$$

Where k_1_ (min^-1^) and k_2_ (g mg^−1^ min^−1^) are the adsorption rate constants of pseudo-first-order and pseudo-second-order adsorption, respectively, and q_e_ and qt are the amounts of adsorption dye (mg/g) at equilibrium and at time t (min).

Figure 11c-f shows the corresponding linear relationships. Table 3 displayed the computed values for the various kinetic parameters along with the matching correlation coefficients. The pseudo-first-order model produced the high correlation coefficients (R^2^ > 0.99) that were observed. Additionally, it seemed that the values of qe,_cal_ and the empirically observed values of qe,_exp_ were rather near. The kinetic data can be effectively characterized by the pseudo-first-order model, as suggested by these results^47^. The computed equilibrium adsorption capacities (qe,_cal_) differed significantly from the experimental values (qe_,exp_). With correlation coefficients of roughly 0.99, the pseudo-second-order model suited the experimental data somewhat, but not very well. These findings suggest that the kinetic data can be adequately described by the pseudo-first-order model.

**Table 3:** Pseudo-first-order, pseudo-second-order, and intra-particle diffusion model kinetic parameters for the adsorption of (**S**_**1**_**)** 20% PMMA -10%CMS-70%- D_400_-MMT “S_1”_, (**S**_**2**_**)** NiWO_4_@ 20% PMMA -10%CMS-70%- D_400_-MMT, and ((**S**_**3**_**)** LaZnFe_2_O_4_@ NiWO_4_@ CMS-MMA @70% wt D_400_-MMT catalyzed systems against XO and R.B dye.

Models parameters	Type of pollutant	Models parameters	S_1_	S_2_	S_3_
Pseudo-first order	Xyelenol Orange	R^2^	0.998	0.995	0.995
		K_1_ (min^−1^)	2.23 × 10^−2^	3.7 × 10^−2^	4.03 × 10^−2^
		Qe,_exp_ (mg/g)	37.00	41.14	44.5
		Qe,_cal_ (mg/g)	34.30	39.80	42.94
Pseudo-second order		R^2^	0.9661	0.9916	0.9960
		K_2_ (g mg min^−1^)	8.09 × 10^−4^	18.23 × 10^–4^	24.33 × 10^−4^
		Qe (mg/g)	45.87	48.08	46.95
Intra-particle diffusion Step I		R^2^	0.9739	0.9522	0.9861
		Ki (mg g^−1^ min^−0.5^)	3.1249	4.4462	5.6763
Step II		Ki (mg g^−1^ min^−0.5^)	4.003	3.023	2.506
Step III		Ki (mg g^−1^ min^−0.5^)	0.1646	0.0082	0.023
Pseudo-first order	Rhodamine B	R^2^	0.9905	0.9962	0.9893
		K_1_ (min^−1^)	2.787 × 10^−2^	3.13 × 10^−2^	3.34 × 10^−2^
		Qe,_exp_ (mg/g)	40.64	43.58	46.52
		Qe,_cal_ (mg/g)	39.16	39.9	39.22
Pseudo-second order		R^2^	0.9894	0.9945	0.9969
		K_2_ (g mg min^−1^)	1.36 × 10^−3^	1.824 × 10^−3^	2.150 × 10^−3^
		Qe (mg/g)	44.05	46.30	48.54
Intra-particle diffusion Step I		R^2^	0.9999	0.9979	0.9945
		Ki (mg g^−1^ min^−0.5^)	5.1047	2.2230	0.1887
Step II		Ki (mg g^−1^ min^−0.5^)	6.621	1.5304	0.1059
Step III		Ki (mg g^−1^ min^−0.5^)	5.8853	1.7102	0.1245

### Adsorption isotherms

**Figure 12:**
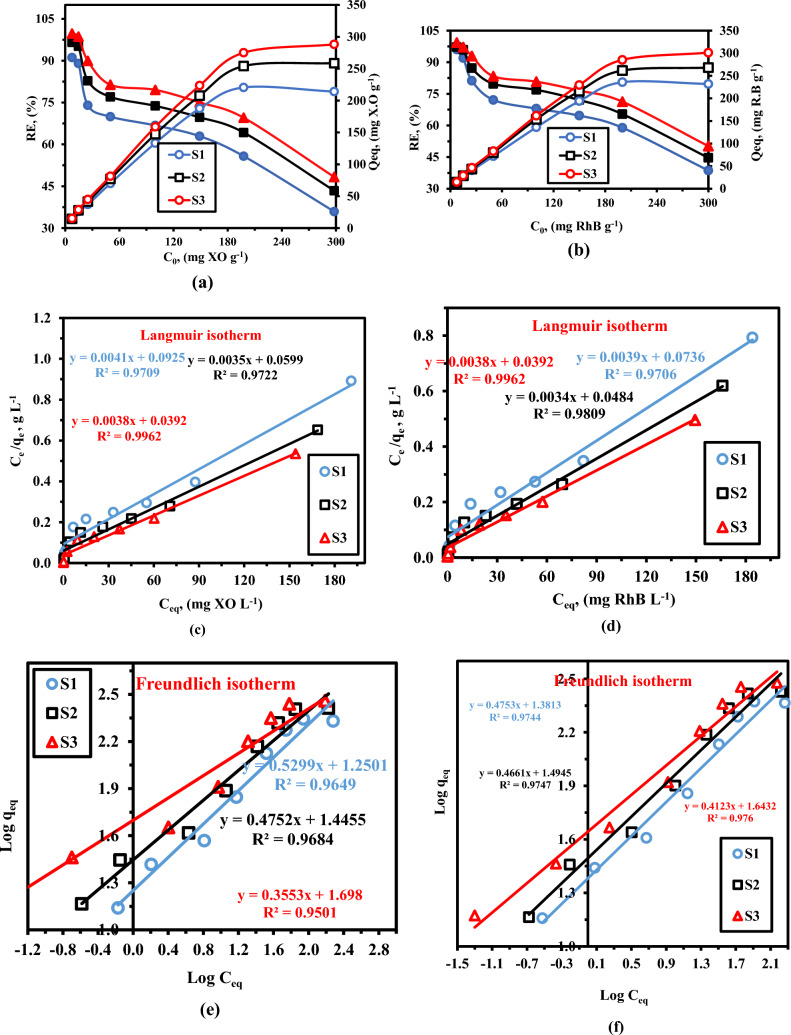
(**a**-**f**). Explain the amount of dye a nanocomposite of “S_1_”, “S_2_”and “S_3_” can bind to (**a**) XO. and (**b**) RhB. Experiments on adsorption: sample dose: 0.025 g/50 mL; pH: 4.0 for XO. and pH: 8.0 for RhB; temperature: 25 °C; equilibrium time: 220 min.

The equilibrium adsorption isotherms are another crucial method for comprehending the mechanism of adsorption, together with adsorption kinetics. Adsorption isotherms showed a link between the concentration of dye in the liquid phase at a constant temperature and the quantity of dye adsorbed per unit mass of the sorbent. The adsorption isotherms, as shown in Fig. 12a,b. can be used to highlight the correlations between the equilibrium concentration of XO and RhB in solution and the XO and RhB adsorption capacity of LaZnFe_2_O_4_@NiWO4@D400-MMT@CMS/MMA nanocomposites. Figure 12a,b, depicts the impact of various initial dye solution concentrations on the generated nanocomposite’s ability for adsorption. The initial dye concentration plays a significant role in determining the nanocomposite’s ability to adsorb. According to Fig. 12a,b, the adsorption capabilities of the nanocomposite grew significantly from 15.1 mg/g to 288.07 mg/g when the concentration of xylenol orange(XO) (pH 4.0) and rhodamine B dye (RhB) (pH 8.0) was increased from (7.5 mg/L to 300 mg/L) for 220 min of adsorption, and barely increased with further dye concentration increase.The experimental data in Fig. 12c-f were further analyzed using the Langmuir and Freundlich isotherm models. The Freundlich adsorption model postulates that adsorption occurs on heterogeneous surfaces^48^, whereas the Langmuir isotherm^49^ is based on the assumption that all adsorption sites are identically homogeneous and that monolayer adsorption occurs on a surface with a finite number of identical adsorption sites. The following is the linear form of the Freundlich and Langmuir isotherms:

Langmuir equation

7 $$q_{e} = \frac{{q_{m} \times b \times C_{e} }}{{{\text{1 + b C}}_{e} }}$$

After integration:

8 $$\frac{{{\text{C}}_{{\text{e}}} }}{{{\text{q}}_{{\text{e}}} }}{ = }\frac{{1}}{{{\text{b}}{\text{. q}}_{{\text{m}}} }}{ + }\frac{{{\text{C}}_{{\text{e}}} }}{{{\text{q}}_{{\text{m}}} }}$$

Freundlich equation:

9 $${\text{q}}_{{{\text{eq}}}} {\text{ = K}}_{{\text{F}}} {\text{C}}_{{\text{e}}}^{{\text{1/n}}}$$

After integration:

10 $${\text{log q}}_{{\text{e}}} {\text{ = log K}}_{{\text{F}}} { + }\frac{{1}}{{\text{n}}}{\text{ log C}}_{{\text{e}}}$$

where C_e_ is the equilibrium concentration (mg L^−1^), q_e_ is the amount absorbed at equilibrium (mg g^−1^), q_m_ is the maximum adsorption capacity of the sorbent (mg g^−1^), b is the Langmuir isotherm constants (L mg^−1^), K_f_ is the Freundlich isotherm constant, and n (dimensionless) is the heterogeneity factor.

The two isotherm models indicated above were thus able to fit the experimental results of XO and RhB equilibrium adsorption on LaZnFe_2_O_4_@NiWO_4_@D400-MMT@CMS/MMA nanocomposites, as illustrated in Fig. 11c-f. The relevant parameters and correlation coefficients were then compiled in Table 4. The adsorption data fit the Langmuir isotherm model (R^2^_**XO**_ = 0.9761,0.9803, 0.9962, and R^2^_**RhB**_ = 0.9706, 0.9809, 0.9962) better than those using the Friedrich isotherm model (R^2^_**XO**_ = 0.9448, 0.9537,0.9424, and R^2^_**RhB**_ = 0.9744, 0.9747, 0.976), indicating that the characteristic of adsorption should be the monolayer adsorption process of XO and RhB on the LaZnFe_2_O_4_@NiWO_4_@D400-MMT@CMS/MMA nanocomposites, with corresponding monolayer-saturated adsorption capacity of 263.16 and 247.09 mg g^−1^ for XO and RhB, respectively^50^**.**

**Table 4:** The adsorption parameters for the Langmuir and Freundlich models’ isotherms.

Sample	Dye	Langmuir			Freundlich		
		R^2^	K_L_ (L g^−1^)	q_m_ (mg g^-1^)	R^2^	K_f_ (mg g^−1^)	1/n
**S**_**1**_	XO	0.9761	0.1144	256.41	0.9448	18.62	0.55
**S**_**2**_	XO	0.9803	0.0969	303.03	0.9537	29.44	0.50
**S**_**3**_	XO	0.9962	0.0666	263.16	0.9424	54.60	0.38
**S**_**1**_	RhB	0.9706	0.5299	13.58	0.9744	24.06	0.47
**S**_**2**_	RhB	0.9809	0.0969	266.12	0.9747	31.22	0.46
**S**_**3**_	RhB	0.9962	0.0702	247.09	0.976	43.97	0.41

Table 5 reviews the assessment of adsorption capacity of XO and RhB dye in the presence of LaZnFe_2_O_4_ supported NiWO_4_@D400-MMT@CMS/MMA nanocomposites with other previous work sample.

**Table 5:** Comparison of the adsorption capacity of XO and RhB dye by various adsorbents.

Adsorbents	Pollutant	q_m_ (mg/g)	References
MIL-101(Cr)	XO	324	Chen et al.^56^
Monodispersed Silica Nanoparticles Incorporated Nanocomposites	XO	1.828	Kaur et al.^4^
Coal Ash	XO	0.74	Mohammad et al.^60^
graphene-based nickel nanocomposite	RhB	65.31	Jinendra et al.^57^
Cal-ZIF-67/AC	RhB	46.2	Li et al.^58^
Graphene	RhB	201.207	Liu et al.^54^
ZnFe_2_O_4_ nanocomposite	RhB	12.1	Konicki et al.^59^
LaZnFe_2_O_4_@NiWO_4_@D_400_-MMT@CMS/MMA nanocomposites	XO	263.16	Current work
	RhB	247.09	

### Effect of temperature and thermodynamic studies

**Figure 13:**
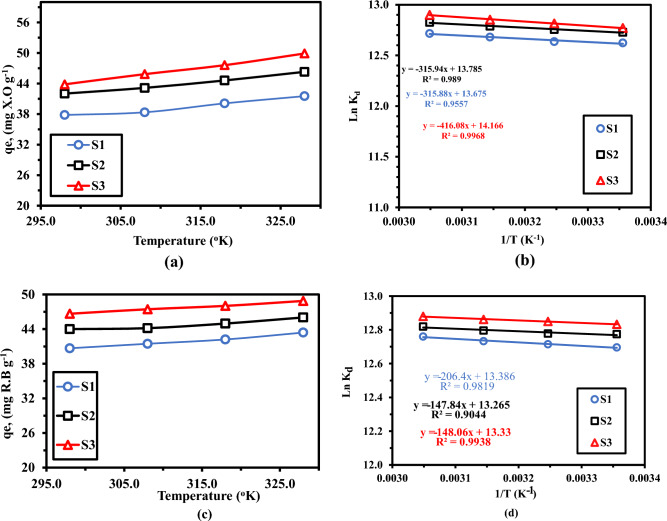
Effect of the temperature on adsorption capacity of “S_1_”, “S_2_” and “S_3_” nanocomposites for (**a,b**) XO, and (**c,d**) RhB. Adsorption experiment /dye concentration: 300 mg/L; sample dose: 0.05 g/100.00 mL; pH: 4.0 for X.O, and 8.0 for RhB; temperature: 30–50 ◦C; equilibrium time: 220 min.

The impact of temperature on the S_1_, S_2_, and S_3_ nanocomposite’s ability was depicted in Fig. 13. As can be shown, as the temperature increased from 25 to 55 °C, the XO dye’s adsorption capacity for S_1_, S_2_, and S_3_ nanocomposites slightly increased from 37.82 mg/g to 41.52 mg/g. The phenomena showed that the XO dye may adhere to the S_3_ nanocomposite more easily at high temperatures. The internal structure of the absorbent may swell as a result of increasing temperature, further allowing the penetrating of the large dye molecule, as is well known^51^**.** However, when temperature increase, the mobility of the large dye molecules increases, which causes a gradual increase in the adsorption capacity of the nanocomposite. RhB obey the same trend as XO., as the temperature increased the dye’s adsorption capacity increased.

Table 6 describes the thermodynamic variables, the absolute H° values are arranged in descending order according to sorption capacity, demonstrating a strong sorption affinity as **S**_**2**_ (− 2.626 kJ mol^−1^) > **S**_**1**_ (− 2.925 kJ mol^-1^) > **S**_**3**_ (− 3.495 kJ mol^-1^). The negative value supports that the reactions have an exothermic character and low temperatures make the process more favorable. The sorption of XO and RhB is assumed to be controlled by physical forces because the H^o^ values are less than 40 kJ mol^−1^ (Ren et al.^52^). The increase in randomness following the contact of XO at the solid–liquid border, as indicated by the positive value of S°. The sorption reaction is thought to be spontaneous based on the negative values of G°, which fall within the same range from -36.806 to − 42.089 kJ mol^-1^.

**Table 6:** The thermodynamic variables of “S_1_” D400-MMT@MMA-CMS, “S_2_” NiWO_4_@D400-MMT@MMA-CMS, and “S_3_” LaZnFe_2_O_4_@NiWO_4_@D400-MMT@MMA-CMS nanocomposites.

Sorbent	dye	Temp., K	∆H^o^	∆S^o^	∆G^o^	T∆S (KJ/mol)	R^2^
S_1_	XO	298	− 2925.53	113.69	− 36,806.33	33,880.80	0.9557
		308			− 37,943.27	35,017.74	
		318			− 39,080.21	36,154.68	
		328			− 40,217.15	37,291.62	
S_2_		298	− 2626.73	114.61	− 36,780.06	34,153.33	0.989
		308			− 37,926.14	35,299.41	
		318			− 39,072.22	36,445.50	
		328			− 40,218.31	37,591.58	
S_3_		298	− 3459.29	117.78	− 38,556.57	35,097.28	0.9968
		308			− 39,734.34	36,275.05	
		318			− 40,912.10	37,452.81	
		328			− 42,089.86	38,630.57	
S_1_	RhB	298	− 1716.01	111.29	− 34,880.79	33,164.78	0.9819
		308			− 35,993.70	34,277.69	
		318			− 37,106.61	35,390.60	
		328			− 38,219.52	36,503.51	
S_2_		298	− 1229.14	110.29	− 34,094.13	32,864.99	0.9044
		308			− 35,196.99	33,967.84	
		318			− 36,299.84	35,070.70	
		328			− 37,402.69	36,173.55	
S_3_		298	− 1230.97	110.83	− 34,257.01	33,026.03	0.9938
		308			− 35,365.26	34,134.29	
		318			− 36,473.52	35,242.55	
		328			− 37,581.77	36,350.80	

Lu et al., 2018, report the relationship between the temperature and the absolute G values. Generally, the following order is supported by the experimental results for the pH effect, kinetics (Tables 3), isotherms (Table 4), and thermodynamics (absolute values of H°, S°, and G° (Table 6)): S_3_ > S_2_ > S_3._

## Mechanism of dye adsorption

PAMAM/CMS and LaZnFe_2_O_4_’s which exfoliated with clay layers appear to promote the stabilization of positively charged RhB molecules over clay-deprotonated silanols. Numerous studies have revealed that the active Si–OH groups in the clay platelets and the dye’s nitrogen atoms engage in hydrogen bonds as well as electrostatic interactions. It’s likely that the LaZnFe_2_O_4_ phase’s attachment to the deeply ingrained amino groups in the jeffamine prevents the generated radicals from interacting with RhB molecules.

Nonetheless, the extreme stability of the polymer colonies appears to produce radicals due to their reducing nature, which is caused by their amide and terminal amine groups. A reducing environment like this could cause LaZnFe_2_O_4_ and NiWO_4_ to function as a “effective battery,” releasing a stream of radicals toward the majority of the solution. Owing to the electrostatic repulsion between the silanols (SiO − H) of clay platelets and the carboxylate groups (COO^−^) of XO, the XO molecules will preferentially remain in the majority of the solution, vulnerable to attack from the generated radicals. As a result of these contact events, dye adsorption will have a notable mineralization efficiency of about 90%.

**Figure 14:**
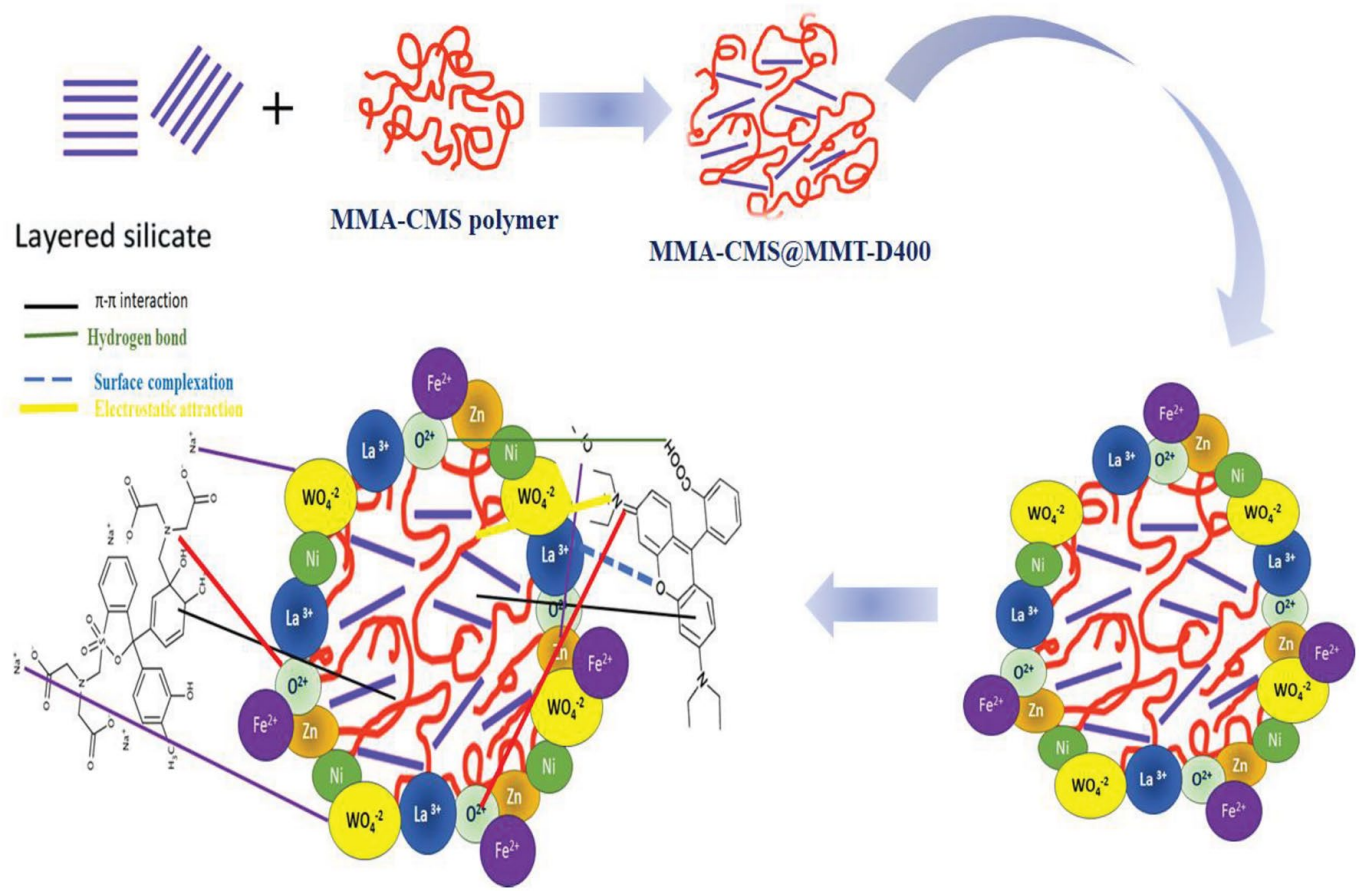
Proposed adsorption mechanism by LaZnFe_2_O_4_ supported NiWO4@D400-MMT@CMS/MMA nanocomposites.

Conversely, LaZnFe_2_O_4_@NiWO_4_@D400-MMT@CMS/MMA’s dominant RhB removal effectiveness is most likely related to the NPs’ anchoring into the interior amine groups of the Jeffamine, which are readily prone to interacting with -C = O molecules in polymers. The negatively charged PMMA@CMS and the anchored LaZnFe_2_O_4_ and NiWO_4_ appear to be the only places where the positively charged RhB molecules tend to site, though. This is likely due to steric barrier between these colonies and the dye quaternary amine-protonated centers (N^+^-)^53^**.** This enables the dye to be readily damaged by the radicals produced, exhibiting an approximately 93% mineralization efficiency. Conversely, the negatively charged XO dye exhibits an electrostatic repulsion tendency with the negatively charged ζ-potential of the LaZnFe_2_O_4_@NiWO_4_@PMMA@CMS nanocomposite, causing the dye to exist in the bulk of solution independently of the radicals’ surroundings. See Fig. 14.

### Desorption studies and reusability Of LaZnFe_2_O_4_ supported NiWO_4_@D400-MMT@CMS/MMA nanocomposites

The recyclable nature of the LaZnFe_2_O_4_-supported NiWO_4_@D400-MMT@CMS/MMA nanocomposites were examined by introducing adsorbents loaded with XO. and RhB into a 0.1 M NaOH solution and subjecting it to ultrasonication following magnetic separation. The starting concentrations of 50 mg/L were used for the investigation. There were five adsorption–desorption cycles carried out^51^**.**

11 $$\user2{\% Desorption} = \user2{ }\frac{{{\varvec{C}}_{{{\varvec{des}}}} }}{{{\varvec{C}}_{{{\varvec{ads}}}} }}\user2{ } \times \user2{ }100$$

where C_ads_ is the dye’s adsorbed concentration and C_des_ is the dye’s desorbed concentration.

**Figure 15:**
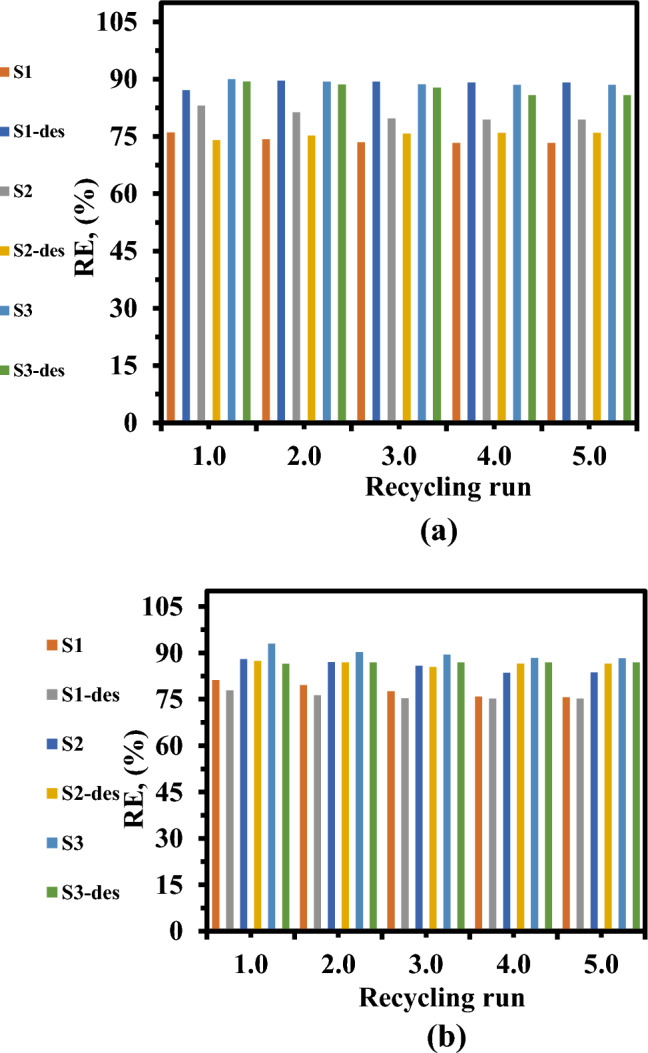
The reusability of S_1_, S_2_, and S_3_ nanocomposites for adsorption of (**a**) XO, and (**b**) RhB.

For XO, the percentage of S_1_, S_2_, and S_3_ nanocomposites that desorb in 0.03 M HCl was found to be approximately 85%, 76%, and 89%, respectively. For RhB, the corresponding percentages of desorption were 75%, 86%, and 86.93 for S_1_, S_2_, and S_3_ nanocomposites,respectively. In the instance of XO, adsorption efficiency was determined to be excellent in the first three cycles. The percentage of dye removal employing nanocomposite was reduced to 73, 79%, and 88% for S_1_, S_2_, and S_3_ nanocomposites in the fourth and fifth cycles (Fig. 15a), see Table. 7^54^**.**

**Table 7:** Reusability examinations of the LaZnFe_2_O_4_-supported NiWO_4_@D400-MMT@CMS/MMA nanocomposites in five sequential cycles of adsorption–desorption of XO and RhB.

Rounds	1			2			3			4			5		
Sample	S_1_	S_2_	S_3_	S_1_	S_2_	S_3_	S_1_	S_2_	S_3_	S_1_	S_2_	S_3_	S_1_	S_2_	S_3_
X.O removal %	76.03	83.09	90.01	74.25	81.34	89.3	73.46	79.74	88.7	73.32	79.39	88.5	73.32	79.39	88.5
RhB removal %	81.2	88.21	93.01	79.567	87.01	90.3	77.56	85.87	89.43	75.89	83.56	88.34	75.67	83.66	88.32

While for RhB as depicted in Fig. 15b, the results demonstrated that during the course of the five consecutive adsorption–desorption cycles, the removal efficiency of RhB only reduced by 5.53% , 4.4% , and 4.69%, respectively, but during the first two cycles, the decline was just 1%^55^**.**This suggests that NiWO_4_@D400-MMT@CMS/MMA nanocomposites supported by LaZnFe_2_O_4_ have good adsorption and reusability properties, which may have potential uses in the removal of cationic dyes from aqueous solutions without noticeably reducing their ability to remove XO and RhB.

## Conclusions

By using a straightforward thermal process, LaZnFe_2_O_4_@NiWO_4_@D400-MMT@CMS/MMA **“S**_**3**_**”** nanocomposites were successfully created and used to remove dye from an aqueous solution. the LaZnFe_2_O_4_@NiWO_4_@D400-MMT@CMS/MMA nanocomposite showed an adsorption efficiency of around 90% for XO and 93% for RhB with qe > 42.62 mg g^−1^ and 46.26, respectively. To identify the adsorption mechanism, equilibrium isotherms and kinetics of adsorption were investigated. The dose of 0.05 g L-1 was shown to be the ideal amount for adsorption at each pH for XO and RB, with dosages beyond 0.05 g L-1 0.2 not significantly altering the adsorption efficacy. LaZnFe_2_O_4_@NiWO_4_@D400-MMT@CMS/MMA**”S**_**3**_**″ **nanocomposite quickly absorbed dyes, and the X.O. and R.B. adsorption kinetics followed a pseudo-first-order model, pointing to a physisorption mechanism. Furthermore, because intra-particle diffusion offered a good fit to the experimental kinetic data, it is believed that dye molecules had a substantial role in the early stages of adsorption. The results of equilibrium isotherms showed that the behavior of XO and RhB adsorption onto LaZnFe_2_O_4_@NiWO_4_@D400-MMT@CMS/MMA nanocomposite closely matched the Langmuir model. LaZnFe_2_O_4_ @ NiWO4@D400-MMT@CMS/MMA nanocomposites have a five-cycle maximum reuse period before seeing a little performance decrease. The results of the adsorption tests indicated that S_1_, S_2_, and S_3_ nanocomposites had an adsorption capacity towards XO adsorbates of 37, 41.14, and 44.5, respectively, and an adsorption capacity towards c RhB of 40.64, 43.58, and 46.42. The positive value of S° indicates that the increase in randomness happened after the dye came into contact with the solid–liquid interface. According to the negative values of G°, the sorption reaction is thought to happen on its own.

## Data Availability

The author’s data supporting this study’s findings are available upon request. Safaa. R. Fouda who should be contacted if someone wants to request the data from this study.

## References

[1] Sowmya A, Meenakshi S (2014). Effective utilization of the functional groups in chitosan by loading Zn (II) for the removal of nitrate and phosphate. Desalin. Water Treat..

[2] Naushad M, Ahamad T, Alothman ZA, Al-muhtaseb AH (2019). Green and Eco-Friendly Nanocomposite for the Removal Of Toxic Hg(II) Metal ion from Aqueous Environment: Adsorption Kinetics & Isotherm Modelling.

[3] Daneshvar E, Vazirzadeh A, Niazi A (2017). Desorption of methylene blue dye from brown macroalga: Effects of operatingparameters, isotherm study and kinetic modeling. J. Clean. Prod.

[4] Kaur K, Jindal R, Bandhu M (2020). Monodispersed silica nanoparticles incorporated nanocomposites of gelatin and psyllium for sequestration of noxious pollutants. J. Polym. Environ.

[5] Nakata K, Ochiai T, Murakami T, Fujishima A (2012). Photoenergy conversion with TiO_2_ photocatalysis: New materials and recent applications. Electrochim. Acta.

[6] Zhou P, Yu J, Jaroniec M (2014). All-solid-state Z-scheme photocatalytic systems. Adv. Mater..

[7] Adeola AO, Nomngongo NP (2022). Advanced polymeric nanocomposites for water treatment applications: A holistic perspective. Polymers.

[8] Ferfera-Harrar H, Dairi N (2014). Green nanocomposite films based on cellulose acetate and biopolymer-modified nanoclays: Studies on morphology and properties. Iran. Polym. J..

[9] Fadillah G, Yudha SP, Sagadevan S, Fatimah I (2020). Magnetic iron oxide/clay nanocomposites for adsorption and catalytic oxidation in water treatment applications. Open Chem..

[10] Nawaz HM, Umar M, Maryam R, Nawaz I, Razzaq HT, Malik L (2022). Polymer nanocomposites based on TiO2 as a reinforcing agent: An overview. Adv. Eng. Mater..

[11] Olatunji, M. A., Khandaker, M. U., Amin, Y. M. & Mahmud, H. N. M. E. International conference for innovation in biomedical engineering and life sciences ICIBEL2015. (eds Ibrahim, F. *et al.*), 6–8 December 2015, Putrajaya, Malaysia. p. 30–5 (2016).

[12] Cui Y, Kumar S, Kona BR, Houcke DV (2015). Gas barrier properties of polymer/clay nanocomposites. RSC Adv..

[13] Choudalakis G, Gotsis AD (2009). Permeability of polymer/clay nanocomposites: A review. Eur. Polym. J..

[14] Kotal M, Bhowmick AK (2015). Polymer nanocomposites from modified clays: Recent advances and challenges. Prog. Polym. Sci..

[15] Zhu TT, Zhou CH, Kabwe FB, Wu QQ, Li CS, Zhang JR (2019). Clays and carbon nanotubes as hybrid nanofillers in thermoplastic-based nanocomposites—A review. Appl. Clay Sci..

[16] Khan, W. S., Hamadneh, N. N., Waqar, A. & Khan, W. A. Polymer nanocomposites: Synthesis techniques, classifcation and properties. *Sci. Appl. Tailor. Nanostruct.* 50–66 (2016).

[17] Wolf C, Angellier-Coussya H, Gontarda N, Doghierib FG, vol,  (2018). How the shape of fillers affects the barrier properties of polymer/non-porous particles nanocomposites: A review. J. Membr. Sci..

[18] Merinska D, Kubisova H, Kalendova A, Svoboda P, Hromadkova J (2011). Processing and properties of polyethylene/montmorillonite nanocomposites. J. Thermoplast. Compos. Mater..

[19] Cui Y (2015). Gas barrier properties of polymer/clay nanocomposites. RSC Advances.

[20] Nordqvist D, Hedenqvist MS (2009). Transport Properties of Nanocomposites Based on Polymers and Layered Inorganic Fillers.

[21] Petronella F, Truppi A, Ingrosso C, Placido T, Striccoli M, Curri ML, Agostiano A, Comparelli R (2017). Nanocomposite materials for photocatalytic degradation of pollutants Catal. Today.

[22] Mandal, D. Denitration of drinking water and nitrate-containing effluents, physical chemical and biological treatment processes for water & wastewater. 1–8 (Nova Science Publishers, Inc., 2015).

[23] Zhang Y (2014). Microstructure and microwave dielectric properties of MgNb_2_O_6_–ZnTa_2_O_6_ composite ceramics prepared by layered stacking method. J. Mater. Sci. Mater. Electron.

[24] Ahmadzadeh M, Almasi-Kashia M, Ramazani A (2015). Preparation and characterization of the magnesium aluminate nanoparticles via a green approach and its photocatalyst application. J. Nanostruct..

[25] Ghoreishi FS, Ahmadi V, Samadpourc M (2013). Preparation and characterization of cadmium titanate nanoparticles via novel sol–gel method and its photocatalyst application. J. Nanostruct..

[26] Panahi-Kalamuei M, Mousavi-Kamazani M, Salavati-Niasari M (2014). Facile hydrothermal synthesis of tellurium nanostructures for solar cells. J. Nanostruct..

[27] Ruhollah T, Mahdi N, Saman R (2016). Synthesis, characterization and optical properties of lanthanum doped zinc ferrite nanoparticles prepared by sol–gel method. J Mater Sci.

[28] Rahimi-Nasarabadi M (2014). Electrochemical synthesis and characterization of zinc sulfide nanoparticles. J. Nanostruct..

[29] Khosravifard E, Salavati-Niasari M, Dadkhah M, Sodeifian G (2010). Synthesis and characterization of TiO2-CNTs nanocomposite and investigation of viscosity and thermal conductivity of a new nanofluid. J. Nanostruct..

[30] Jose R, Thavasi V, Ramakrishna S (2009). Metal oxides for dye-sensitized solar cells. J. Am. Chem. Ceram. Soc..

[31] Brusentsov NA, Gogosov V, Magn J, Mater. Magn.  (2001). Evaluation of ferromagnetic fluids and suspensions for the site-specific radiofrequency-induced hyperthermia of MX11 sarcoma cells in vitro. J. Magn. Magn..

[32] Buonsanti R (2010). Architectural control of seeded-grown magnetic-semicondutor iron oxide-TiO_2_ nanorod heterostructures: The role of seeds in topology selection. J. Am. Chem. Soc..

[33] Atif M, Hasanain SK, Nadeem M (2006). Magnetization of sol–gel prepared zinc ferrite nanoparticles: Effects of inversion and particle size. Solid State Commun..

[34] Tholkappiyan R, Vishista K (2014). Influence of lanthanum on the optomagnetic properties of zinc ferrite prepared by combustion method. Phys. B Condens. Matter.

[35] Jonghun H, Byung-Moon J, Jiyong H, Gooyong L, YeominChang YMP (2019). Highly efficient organic dye removal from waters by magnetically recoverable La_2_O_2_CO_3_/ZnFe_2_O_4_-reduced graphene oxide nanohybrid. Ceram. Int..

[36] Toledo-Antonio JA, NavaMart´ınezBokhimi NMX (2002). Correlation between the magnetism of non-stoichiometric zinc ferrites and their catalytic activity for oxidative dehydrogenation of 1-butene. Appl. Catal..

[37] Deng Y, Zhang Q, Tang S, Zhang L, Deng S, ShibcChen ZG (2011). One-pot synthesis of ZnFe2O4/C hollow spheres as superior anode materials for lithium-ion batteries. Chem. Commun..

[38] Casbeer E, Sharma VK, Li X-Z (2012). Synthesis and photocatalytic activity of ferrites under visible light: A review. Sep. Purif. Technol..

[39] Li X, Hou Y, Zhao Q, Teng W, Hu X, Chen G (2011). Capability of novel ZnFe_2_O_4_ nanotube arrays for visible-light induced degradation of 4-chlorophenol. Chemosphere.

[40] Sabbagh F (2019). Mechanical properties and swelling behavior of acrylamide hydrogels using montmorillonite and kaolinite as clays. J. Environ. Treat. Tech..

[41] Cao XB, Gu L, Lan XM, Zhao C, Yao D, Seng WJ (2007). Synthesis, characterization and optical properties of lanthanum doped zinc ferrite nanoparticles prepared by sol–gel method Mater. Chem. Phys..

[42] Cao SW, Zhu YJ, Cheng GH, Huang YH (2009). Adsorption kinetic and equilibrium studies for methylene blue dye by partially hydrolyzed polyacrylamide/cellulose nanocrystalnanocomposite hydrogels. J. Hazard. Mater..

[43] Zhou C, Wu Q, Lei T, Negulescu II (2014). Adsorption kinetic and equilibrium studies for methylene blue dye by partially hydrolyzed polyacrylamide/cellulose nanocrystal nanocomposite hydrogels. Chem. Eng.

[44] Wang L, Wang A (2008). Adsorption behaviors of Congo red on the N, O-carboxymethyl-chitosan/montmorillonite Nanocomposit*e*. Chem. Eng..

[45] Fouda SR, El-Sayed IE, Attia NF, Abdeen MM, Abdel AH, Aleem A, Nassar I, Mira HI, Gawad EA, Kalam A, Al-Ghamdi AA, Galhoum AA (2022). Mechanistic study of Hg(II) interaction with three different α-aminophosphonate adsorbents: Insights from batch experiments and theoretical calculations. Chemosphere..

[46] Sun SL, Wang AQ (2006). Adsorption kinetics of Cu(II) ions using N, Ocarboxymethyl- chitosan. J. Hazard. Mater..

[47] Saber-Samandari S, Gazi M (2015). Pullulan based porous semi-IPN hydrogel: Synthesis, characterization and its application in the removal of mercury from aqueous solution. J. Taiwan. Inst. Chem. E..

[48] Saber-Samandari S, Gazi M (2013). Removal of mercury (II) from aqueous solution using chitosan-graftpolyacrylamide semi-IPN hydrogels. Separ. Sci. Technol..

[49] Langmuir I (1918). The adsorption of gases on plane surfaces of glass, mica and platinum. J. Am. Chem. Soc..

[50] Freundlich, H. M. F. Uber die adsorption in lasungen. *Z. Phys. Chem.* **57**, 385–470 (1906).

[51] Singh SA, Vemparala B, Madras G (2015). Adsorption kinetics of dyes and their mixtures with Co_3_O_4_-ZrO_2_ composites. J. Environ. Chem. Eng..

[52] Ren Y, Liu G, Pu G, Chen Y, Chen W, Shi L (2020). Spatiotemporal evolution of the international plastic resin trade network. J. Cleaner Prod..

[53] Daneshvar E, Vazirzadeh A, Niazi A (2017). Desorption of methylene blue dye from brown macroalga: efects of operatingparameters, isotherm study and kinetic modeling. J. Clean. Prod..

[54] Liu H, Sun R, Feng S, Wang D, Liu H (2019). Rapid synthesis of a silsesquioxane-based disulfide-linked polymer for selective removal of cationic dyes from aqueous solutions. Chem. Eng. J..

[55] Gang X, Wusong X, Shuai T, Wenjie Z, Tian Y, Yuxiao Wu, Qizhong X, Yusef KK, Hongjian G (2021). Enhanced phosphate removal from wastewater by recyclable fiber supported quaternary ammonium salts: Highlighting the role of surface polarity. Chem. Eng. J..

[56] Chen C, Zhang M, Guan Q, Li W (2012). Kinetic and thermodynamic studies on the adsorption of xylenol orange onto MIL-101(Cr). Chem. Eng. J..

[57] Jinendra U, Bilehal D, Nagabhushana BM, Kumar AB (2021). Adsorptive removal of Rhodamine B dye from aqueous solution by using graphene–based nickel nanocomposite. Heliyon.

[58] Li Y, Yan X, Hu X, Feng R, Zhou M (2019). Trace pyrolyzed ZIF-67 loaded activated carbon pellets for enhanced adsorption and catalytic degradation of Rhodamine B in water. Chem. Eng. J..

[59] Konicki W, Siber D, Narkiewicz U (2017). Removal of Rhodamine B from aqueous solution by ZnFe_2_O_4_ nanocomposite with magnetic separation performance. Pol. J. Chem. Technol..

[60] Mohammad I, Khalid S, Imtiaz A, Sirraj S (2014). Coal ash as a low cost adsorbent for the removal of xylenol orange from aqueous solution. Iran. J. Chem. Chem. Eng..

